# Establishing Analytical Performance Criteria for the Global Reconnaissance of Antibiotics and Other Pharmaceutical Residues in the Aquatic Environment Using Liquid Chromatography-Tandem Mass Spectrometry

**DOI:** 10.1155/2018/7019204

**Published:** 2018-06-04

**Authors:** Luisa F. Angeles, Diana S. Aga

**Affiliations:** Department of Chemistry, The State University of New York, Buffalo, NY 14260, USA

## Abstract

The occurrence of antibiotics in the environment from discharges of wastewater treatment plants (WWTPs) and from the land application of antibiotic-laden manure from animal agriculture is a critical global issue because these residues have been associated with the increased emergence of antibiotic resistance in the environment. In addition, other classes of pharmaceuticals and personal care products (PPCPs) have been found in effluents of municipal WWTPs, many of which persist in the receiving environments. Analysis of antibiotics by liquid chromatography-tandem mass spectrometry (LC-MS/MS) in samples from different countries presents unique challenges that should be considered, from ion suppression due to matrix effects, to lack of available stable isotopically labeled standards for accurate quantification. Understanding the caveats of LC-MS/MS is important for assessing samples with varying matrix complexity. Ion ratios between quantifying and qualifying ions have been used for quality assurance purposes; however, there is limited information regarding the significance of setting criteria for acceptable variabilities in their values in the literature. Upon investigation of 30 pharmaceuticals in WWTP influent and effluent samples, and in receiving surface water samples downstream and upstream of the WWTP, it was found that ion ratios have higher variabilities at lower concentrations in highly complex matrices, and the extent of variability may be exacerbated by the physicochemical properties of the analytes. In setting the acceptable ion ratio criterion, the overall mean, which was obtained by taking the average of the ion ratios at all concentrations (1.56 to 100 ppb), was used. Then, for many of the target analytes included in this study, the tolerance range was set at 40% for WWTP influent samples and 30% for WWTP effluent, upstream, and downstream samples. A separate tolerance range of 80% was set for tetracyclines and quinolones, which showed higher variations in the ion ratios compared to the other analytes.

## 1. Introduction

In recent years, studies have reported the occurrence of pharmaceuticals and personal care products (PPCPs), including antibiotics and selective serotonin reuptake inhibitors (SSRIs), in the environment [1–6]. These drugs are being released through different routes, such as discharges from wastewater treatment plant (WWTP) effluents to surface water, where hospitals and private households contribute a large volume of antibiotics and other pharmaceuticals [2, 7–9]. The presence of PPCPs in effluents of WWTPs in different geographical regions has been documented, with concentrations reported as high as about 125 *µ*g/L [10]. In Germany, the environmental concentration in municipal sewage that comes from the discharge of antibiotics from hospitals and households is predicted to be about 71 mg/L annually [9]. The presence of high levels of pharmaceuticals in the environment has a wide range of ecological effects; for instance, antibiotics may contribute to the development of antibiotic resistance in bacteria due to selective pressure, which is a threat to global health [9, 11, 12].

Analysis of PPCPs in environmental samples is typically performed using liquid chromatography-tandem mass spectrometry (LC-MS/MS) to quantify pharmaceutical concentrations based on triple quadrupole MS [3, 6, 13]. The high selectivity and sensitivity obtained using triple quadrupole MS is achieved when performing selected reaction monitoring (SRM), where a precursor ion is isolated from the first quadrupole and fragmented in the collision cell, followed by isolating selected product ions in the third quadrupole. However, despite this high selectivity, there is still a possibility that a compound other than the target analyte will produce a signal that has a similar *m*/*z* value to either the qualifying ion or the quantifying ion at the same retention time [14], resulting in a significant deviation in the expected ion ratio for the selected fragment ions being monitored by the two SRM transitions.

In order to confirm the presence of a compound, the chromatographic peak must have both the quantitative and qualitative ion transitions with retention times matching those of the standard analyte. In addition, the ion ratio of the two SRM transitions has been used as an additional confirmation criterion, as stated in some legal documents from different organizations such as the European Union (EU) and the US Food and Drug Administration (US FDA), which provide guidelines for the analysis of official samples [15–19]. Having this additional criterion is important since LC-MS/MS has now become the mandatory technique for the analysis of official samples that are used for establishing legal policies [15–19]. Monitoring the ion ratios will provide improved confidence in reporting analyte concentrations, avoiding false positives and false negatives, which have been reported in the literature [14].

Different legal guidelines are currently available from the United Nations (UN), the EU, and the United States of America (USA). The UN set the ion ratio tolerance to be ±20% [16] for the testing of illicit drugs in seized materials and biological specimens. The European Commission Decision (2002/657/EC) requires a tolerance of ±20% to ±50% for the ion ratio, depending on the ion intensities [17], for analytical methods that are used for the testing of official samples in control laboratories. The European Workplace Drug Testing Society sets it at ±20% [19]. The US Department of Agriculture requires a ±20% tolerance in the ratio of the ion transitions [18], while the US FDA sets an ion ratio tolerance of ±20% and ±30% if 2 and 3 diagnostic ions are being monitored, respectively [15]. The weakness of these guidelines, however, is that they are not based on experimental data and are arbitrarily assigned.

Recent studies [20, 21] have been published on performance criteria for the analyses of pesticides in fruits and vegetables and veterinary drugs in biological matrices. For pesticides, a tolerance range of ±20% was established for all compounds at all concentrations, except when one or both product ions have an S/N of 3–15, in which case, a range of ±45% was set. For veterinary drugs, a fixed tolerance range of ±50% for all the compounds at all concentrations was set after evaluation of the ion ratios in different matrices such as muscle, urine, milk, and liver [20, 21]. However, these tolerance values cannot be used for PPCPs because the variability of ion ratios differs per compound and the nature of the sample matrix. This variability is due to differences in the ionization behavior of analytes and the extent of matrix effects. It is not unexpected to observe different effects on the ion ratios of the analytes in wastewater and surface water matrices because the composition of the interferences in environmental samples is different relative to biological samples.

Establishing performance criteria is important because it minimizes the occurrence of false-positive and false-negative detections. In fact, a doubling of false-positive detections was reported without the application of the ion ratio criterion in the analysis of veterinary drugs in the muscle, urine, milk, and liver [14]. Most published and existing methods do not mention the use of any ion ratio criteria [3, 6, 13]. In the US Environmental Protection Agency (EPA) Method 1694 for the determination of PPCPs in environmental samples by LC-MS/MS, the presence of a compound in a sample extract is confirmed when the signal-to-noise ratio (S/N) of the fragment ion of the compound is greater than or equal to 2.5 and its retention time is within ±15 seconds of the calibration verification standard. If these criteria are not met, then an experienced analyst must confirm the presence or absence of a compound [22]. Additionally, in the EPA Method 542, which is for the analysis of PPCPs in drinking water, the acceptable retention time window for the compounds in a sample is within 3 standard deviations for a series of injections. Quality control for this method involves the confirmation of the presence of the quantifying ion of the internal standard and requires that it must be within ±50% of the average area measured in the initial calibration [23]. No criteria regarding the ion ratios have been mentioned in both EPA methods. The absence of quality control measures in published methods may be due to the lack of suitable guidelines in the literature. In order to determine an appropriate tolerance value for the ion ratios, variabilities resulting from the physicochemical nature of the analytes should be investigated at high and low concentrations. The variability in the signal intensities of the qualifier ions is expected to be more significant than that of the quantifier ions because of the relatively lower signals for the qualifier ions.

The aim of this study is to validate and provide guidelines on the use of ion ratios as a criterion for quality control in reporting concentrations of PPCPs in wastewater and surface water samples with varying complexity. To achieve this goal, the ion ratios of 30 PPCPs in different matrices were determined at different concentrations in order to determine a tolerance value that is sufficient to eliminate false positives and false negatives. The matrices studied were WWTP influent and effluent samples and surface water samples from upstream and downstream of the WWTP discharge point collected from the US, Sweden, Switzerland, Hong Kong, and the Philippines, allowing the set tolerance levels to be robust, given that the composition of water samples varies significantly in different parts of the world. The data obtained from these analyses were the basis for the construction of a more accurate and reliable ion ratio criterion which takes into account the differences in the properties of compounds at different concentrations.

## 2. Materials and Methods

Acetaminophen (ACT), acetylsulfamethoxazole (ASMX), azithromycin (AZI), caffeine (CAF), carbamazepine (CBZ), clarithromycin (CLA), enrofloxacin (ENRO), erythromycin (ERY), iopamidol (IOPA), norfloxacin (NOR), oxytetracycline (OTC), sarafloxacin (SARA), sulfachloropyridazine (SCP), sulfadiazine (SPD), sulfadimethoxine (SDM), sulfamerazine (SMR), sulfameter (SMT), sulfamethazine (SMZ), sulfamethizole (SMI), sulfamethoxazole (SMX), tetracycline (TC), and trimethoprim (TMP) were purchased from Sigma-Aldrich. Ciprofloxacin (CIP) and diclofenac (DIC) were obtained from Cambridge Isotope Laboratories, Inc. (Tewksbury, MA). Sulfathiazole was purchased from ICN Biomedicals, Inc. (Irvine, CA). Carbamazepine-d10 (d10-CBZ) was purchased from CDN Isotopes (Quebec, Canada). Chlortetracycline (CTC) was obtained from Acros Organics (VWR International, Westchester, PA). Paroxetine maleate (PRX) and venlafaxine (VEN) were obtained from Cerilliant (Sigma-Aldrich, St Louis, MO). The Barnstead NANOpure™ DIamond (Waltham, MA) purification system was used to obtain 18.2 MΩ water. LC-MS grade methanol and acetonitrile were obtained from EMD Millipore Corporation (Billerica, MA), and formic acid (88%) was purchased from Fisher Chemical (Pittsburgh, PA). Oasis™ HLB solid-phase extraction (SPE) cartridges were purchased from Waters (Milford, MA).

### 2.1. Sample Preparation

Wastewater and surface water samples (0.5 L) were collected in amber glass bottles which were pre-rinsed with 10% nitric acid. The samples were acidified to about pH 2.5 using 40% phosphoric acid and then passed through 0.45 *µ*m glass microfiber filters to remove microorganisms and particulate matter. Then, 2 mL of Na_2_EDTA (5% *w*/*v* in water) was added to each sample. The samples were then spiked with surrogate standards (50 *µ*L of 1000 *µ*g/L surrogate mix solution).

The samples were passed through Oasis HLB SPE cartridges (500 mg, 6 cc) for cleanup and concentration. The SPE cartridges were first conditioned with 6 mL acetonitrile, followed by 6 mL NANOpure water, before the water samples were loaded at a rate of approximately 3–5 mL/min. After loading, the cartridges were dried by keeping them on the SPE manifold with the vacuum on. Then, the SPE cartridges were wrapped in aluminum foil, stored in Ziploc® bags, and shipped with ice to the University at Buffalo for elution and LC-MS/MS analysis. Once received, the samples were eluted using 8 mL of acetonitrile and then dried under N_2_ gas at 35°C. The samples were then spiked with 100 ppb of the internal standard, carbamazepine-d10, in order to account for possible differences in measurements in-between injections due to variations caused by the instrument.

### 2.2. LC-MS/MS Analysis

A Waters Cortecs™ C18^+^ column (Milford, MA) with dimensions 2.1 × 150 mm and 2.7 *µ*m particle size was used for the separation of the 30 PPCPs. Analysis was performed using an Agilent 1200 LC system (Palo Alto, CA) and a Thermo Scientific TSQ Quantum Ultra triple quadrupole MS (Waltham, MA) equipped with a heated electrospray ionization (HESI) probe, operated under positive ionization mode. Timed-SRM mode transition was performed, and the SRM transitions used for the compounds are shown in Table S1.

The mobile phase used for the separation consisted of aqueous 0.3% formic acid (A), and 75% methanol and 25% acetonitrile (B). The gradient began with 90% A and 10% B for three minutes and is ramped up linearly to 100% B for 22 min; this condition was kept for 5 min before it was switched back to 90% A, where it was maintained for 14 min to allow for column equilibration. The flow rate was set at 0.2 mL/min, and the total run time was 45 min.

The spray setting used for the MS was as follows: spray voltage 3000 V, ion sweep gas pressure 0 arbitrary units, vaporizer temperature 350°C, sheath gas pressure 40 arbitrary units (N_2_), auxiliary gas pressure 35 arbitrary units (N_2_), capillary temperature 325°C, collision gas pressure 1.5 mTorr (Ar), cycle time 0.300 s, and Q1 peak width 0.70 FWHM.

### 2.3. Design of the Study

#### 2.3.1. Assessment of Ion Ratio Behavior of PPCPs across Varying Concentrations

A total of 30 PPCPs were studied for the development of an ion ratio criterion (Table 1). A mixture of all the native PPCP standards was prepared using the starting mobile phase of the LC-MS/MS method as the solvent. An initial solution of 100 ppb (*µ*g/L) was made, and then it was serially diluted to obtain mixtures with concentrations of 50, 25, 12.5, 6.25, 3.13, and 1.56 ppb. These standards were analyzed by LC-MS/MS, with nine replicates for each concentration, to obtain the areas of both the quantifying and qualifying ions. The ion ratios were calculated by dividing the area of the quantifying ion by the area of the qualifying ion for each analyte. The average ion ratio and the deviations from the average value for the ion ratios were calculated at all concentrations for each compound.

#### 2.3.2. Assessment of Ion Ratio Behavior of Pharmaceuticals in the Matrix

Samples from WWTP influents and effluents and from receiving surface waters upstream and downstream of the WWTPs were collected from selected sites in five countries: Central, Hong Kong; Manila, Philippines; Vastergotland, Sweden; Zurich, Switzerland; and Virginia, US. The samples from the Philippines were collected in December 2016, while the others were collected in June or July 2016. The exact names and locations of the WWTPs cannot be disclosed as part of the agreement with the WWTP operations. A total of 19 samples were each spiked with 1.56 ppb, 12.5 ppb, 25 ppb, and 100 ppb of the native standard mix and were analyzed by LC-MS/MS to determine the mean ion ratios and standard deviations from the mean for each compound. The variabilities of the ion ratios in the different sample matrices were then evaluated and compared with the values observed in the standards.

#### 2.3.3. Optimization of the Ion Ratio Criterion

The proposed formula to be used in order to optimize the appropriate ion ratio tolerance that will give the least false negative is a mean ion ratio and a tolerance range that will account for variations in the sample matrix. This tolerance range should not be too wide so as to avoid having false positives. To determine the optimum tolerance level, different values were tested for all compounds; the same test was also used to determine whether a single tolerance range would be used for all the matrices or if different ones should be used for wastewater and for surface water. To check the appropriateness of the selected tolerance values, the number of false negatives will be determined using the water extracts spiked with known amounts of standards.

## 3. Results and Discussion

### 3.1. Ion Ratio Variability at Different Concentrations

A total of 30 PPCPs, which include 23 antibiotics, were studied for the development of an ion ratio criterion (Table 1). The classes of antibiotics that were included in this study were sulfonamides, macrolides, quinolones, and tetracyclines.

**Figure 1:**
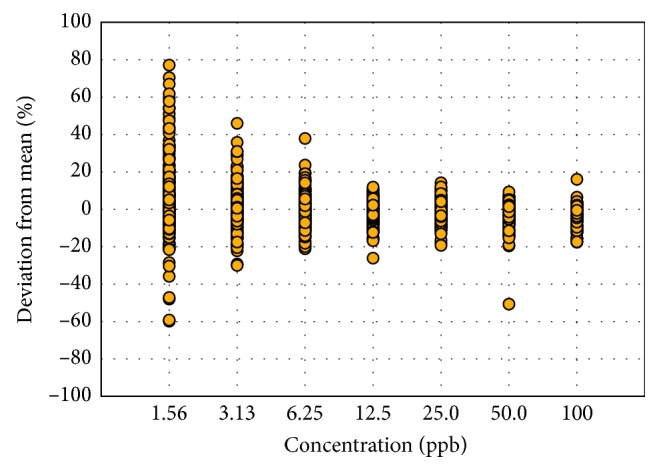
Deviation of ion ratios from the overall mean across different concentrations of 23 PPCPs without tetracyclines and quinolones.

First, the ion ratio behavior of the compounds was studied at different concentrations by analyzing nine replicates of the standard solutions of 1.56, 3.13, 6.25, 12.5, 25.0, 50.0, and 100 ppb in the LC-MS/MS. The overall mean, which is the average ion ratio of all nine replicates at all concentrations, was obtained for each compound (Table S2). The relative percent deviation was then calculated by subtracting the overall mean from each of the data points and then dividing by the overall mean. These values were then plotted against the seven concentrations to see how the ion ratios at each concentration vary from the overall mean of each compound, as shown in Figure 1. The trend for all the compounds is that the variation is highest at the lowest concentration. The average relative standard deviation for all the compounds at 1.56 ppb was 18%, while that for compounds at 100 ppb was only 4%. These results indicate that the differences in the ion ratios at different concentrations should be taken into account because if only one tolerance limit is applied across all concentrations, it is likely that false-negative results will occur at low concentrations, especially at concentrations between 1.56 to 12.5 ppb.

**Figure 2:**
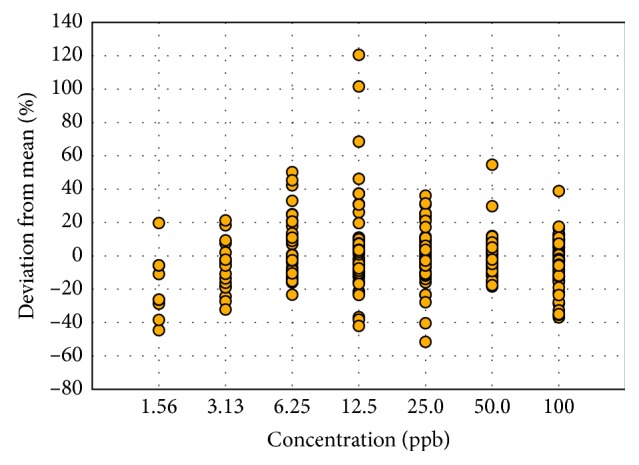
Deviation of ion ratios of tetracyclines and quinolones from the overall mean across different concentrations.

The general trend in the ion ratios for 23 PPCPs is shown in Figure 1; a separate plot for tetracyclines and quinolones was prepared (Figure 2) because the variabilities in the ion ratios were notably higher in these classes of antibiotics than the rest of the PPCPs. Data points at lower concentrations of some compounds were removed in cases where the qualitative ion was not detectable. For example, oxytetracycline was not detected below 25 ppb, chlortetracycline and tetracycline were not detected below 12.5 ppb, sarafloxacin and norfloxacin were not detected below 6.25 ppb, and enrofloxacin was not detected at 1.56 ppb. Therefore, a separate chart (Figure 2) was created for tetracyclines and quinolones since they do not follow the same behavior as the other pharmaceuticals. Based on these data, a separate ion ratio tolerance range is needed for tetracyclines and quinolones in order to capture the wide variations, without affecting the other compounds. It can be observed that the variations in Figure 2 are lower at 1.56 ppb compared to the higher concentrations, but this is because most of these compounds were no longer detected at 1.56 ppb, and these data points were removed in the chart. The deviation from the mean reaches up to 120% at 12.5 ppb for tetracyclines and up to 50% for quinolones at 6.25 ppb. At the highest concentration of 100 ppb, the deviations from the mean in both tetracyclines and quinolones are at 40%, while those for the other PPCPs are only 20%.

**Figure 3:**
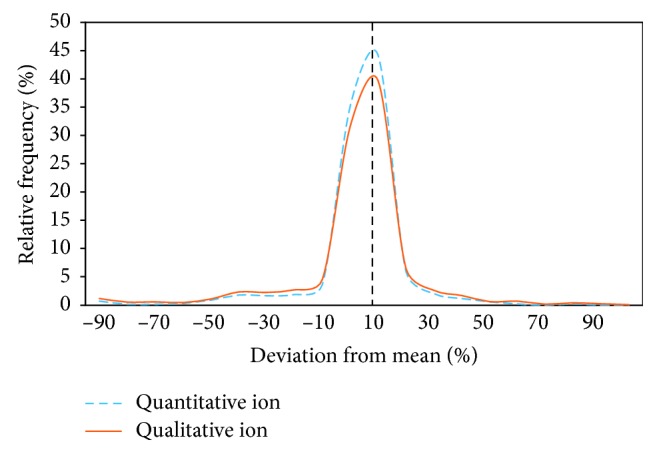
Distribution of the deviation of the quantitative and qualitative ion areas from the mean.

The areas of both the quantitative and qualitative ions were investigated separately in order to identify which of the two ions causes high variations. The deviations in the areas of these ions from the mean were calculated and compared with each other. Since the distribution of variation of both the qualitative and quantitative areas is similar, as seen in Figure 3, this means that both of them contribute equally to the variations, and the ion ratio deviations cannot be attributed to just the quantitative or qualitative ion alone.

### 3.2. Ion Ratio Variability in Wastewater and Surface Water Matrices

A total of 19 different samples were spiked with the pharmaceutical standards at 4 concentrations: 1.56, 12.5, 25.0, and 100 ppb, in order to determine how the differences in the nature of the matrices influence the ion ratios.

**Figure 4:**
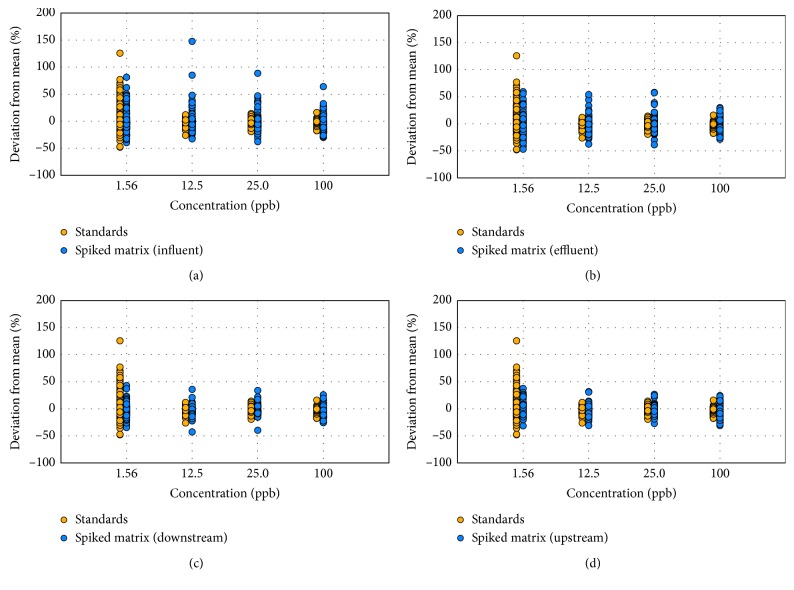
Comparison of ion ratios in spiked matrices and in clean standards. (a) WWTP influent samples; (b) WWTP effluent samples; (c) upstream surface water samples; (d) downstream surface water samples.

Figure 4 shows how the ion ratios change in the influent, effluent, upstream, and downstream water samples in comparison with the clean standard. It can be seen that the variations are higher in the wastewater as compared to those of the surface water samples, with relative standard deviation values of 13%, 11%, 10%, and 9%, for the influent, effluent, downstream, and upstream samples, respectively. This trend was expected since the upstream and downstream samples are less-complex matrices (lower organic matter content than wastewater). It can be seen in the lowest concentration studied (1.56 ppb) that the standards in the clean matrix varied more than the ones spiked in the samples, with relative standard deviations of 18% for the standards and 20%, 14%, 15%, and 12% for the influent, effluent, downstream, and upstream samples, respectively. This is due to the removal of 22 data points at 1.56 ppb because the qualitative ions were no longer detected in the samples.

### 3.3. Optimization of a Tolerance Range for the Ion Ratio Criterion

The formula for the ion ratio criterion that was used is a tolerance range from the mean of each standard compound in the clean matrix. This tolerance range should account for the deviations because of differences in concentrations and the matrix being analyzed. The goal in setting this range is to have the least number of false positives and false negatives. False negatives will occur when the ion ratio of analytes in spiked environmental samples does not meet the tolerance criteria such that the analyte in question will be considered “nondetect.” On the contrary, one cannot set a tolerance range too wide that will likely result in a significant number of false positives. Therefore, a range that will still capture all the variations at the 95% confidence level in both matrices and at different concentrations is needed.

In order to provide an appropriate criterion, the overall mean, which is obtained by taking the average of the ion ratios at all concentrations (1.56 to 100 ppb), will be used. This way, the variations of the ion ratios from low to high concentrations will be taken into account. The tolerance range must then be optimized for the spiked matrices. Tolerance ranges from 10% to 50% were tested to see which one will give the least number of false negatives for each of the water matrices (Table 2). The overall false-negative rate is the weighted average of the percent false negatives for each matrix type.

Since the tetracyclines and quinolones were found to have greater variations than the rest of the PPCPs as seen in Figure 2, a test was performed to check if tetracyclines and quinolones should use a different tolerance level than what is used for the other classes of PPCPs.  Table 3 shows the percentage of false negatives in the samples when the tetracyclines and quinolones were removed. If tetracyclines and quinolones were included, a tolerance range of 50% would give a false-negative result of ≤5%. If removed, a tolerance range of 30% would be enough to give the same value of ≤5% for false negatives.

It is important to have a separate tolerance range for tetracyclines and quinolones because as seen in Table 2, a tolerance range of 50% is needed in order to capture them at the 95% confidence level. This value, however, would be too high for the other PPCPs, where only 30% is required to have the same confidence level (Table 3). Therefore, a fixed tolerance range of 50% for all compounds could potentially result in high false negatives for tetracyclines and quinolones and high false positives for the other PPCPs. Also, at the 50% tolerance range, even if the overall false negatives were already below 5% for many PPCPs, as seen in Table 2, it was observed that the tetracyclines and quinolones still had very high values, with ciprofloxacin having 96% false negatives (Table 4).

For chlortetracycline, ciprofloxacin, enrofloxacin, norfloxacin, and tetracycline, the range needed to be from 70% to 85% in order to have a false-negative rate of ≤5%. Oxytetracycline and sarafloxacin, on the contrary, still have false negatives of up to 29% (downstream) and 20% (upstream), respectively, at a tolerance range of 80% (Table 5). However, setting a wider range may result in greater probability of false positives. Therefore, a tolerance range of 80% was set for the tetracyclines and quinolones, but it is recommended that other criteria such as retention time, peak areas, and the number of points per peak be investigated more carefully in the confirmation of these compounds.

Once the acceptable tolerance range for the mean ion ratio for each analyte was established based on spiked environmental samples, the ion ratio in each sample matrix was also assessed in order to adjust this range accordingly for the influent, effluent, upstream, and downstream samples. The tolerance range that would give ≤5% false negatives was recorded for each matrix type. These values were 40% for the influent samples and 30% for the effluent, upstream, and downstream samples (Table 3). It is expected that the compounds would have higher variations in more complex matrices such as the influent. Since the tolerance range for the influent differed by 10%, it is recommended to establish a different tolerance limit for influent samples to avoid a high false-negative rate in this matrix. If a fixed range of ±20% is used as the tolerance value (Table 2) for all types of matrices, the number of false negatives would be much higher, 26%, 20%, 16%, and 14%, for the influent, effluent, downstream, and upstream samples, respectively. Therefore, it is important to have a separate tolerance range for certain compounds in different environmental matrices.

### 3.4. Applying the Optimized Ion Ratio Criterion in Real Water Samples from around the World

The optimized ion ratio criterion for each of the target PPCPs was applied to real environmental samples that were not spiked with standards. These samples were wastewater influents and effluents and receiving surface waters which are located upstream and downstream of the respective WWTPs, collected from 5 different countries. For influent samples, an analyte is said to be positively detected in the sample if its ion ratio is within the mean ± 40% of the reference standard. For effluent, upstream, and downstream samples, analytes with the mean ion ratio within ±30% of the standards are considered positive detection. Note that the results in Table 6 do not include detections for tetracyclines and quinolones, for which a different tolerance range was set.

The compounds that were detected outside the range were acetylsulfamethoxazole in the effluent samples, azithromycin in the effluent and upstream samples, and clarithromycin in the downstream samples, with the details shown in Table 7.

The compounds with ion ratios that fell outside the set tolerance range were investigated individually to confirm if these were real detections or not by checking the presence of both the quantitative and qualitative ions and if the shift in retention time is not more than 0.5 min. It was found that all of them had both ions, and their retention times were within the acceptable range. Since their calculated ion ratios are still close to the limits of the range, these were still considered as positive detections. In cases like this where the calculated ion ratios are close to the limits of the range and retention times are within the acceptable shift, it is recommended that the qualitative ion be checked to make sure that its signal is at least 3 times that of the noise (Table 6).

A total number of 37 detections for ciprofloxacin, norfloxacin, and tetracycline were found for tetracyclines and quinolones in the samples. The ion ratios of all 37 peaks in all matrices were within the set tolerance range of 80%, and they passed other criteria for peak confirmation.

**Figure 5:**
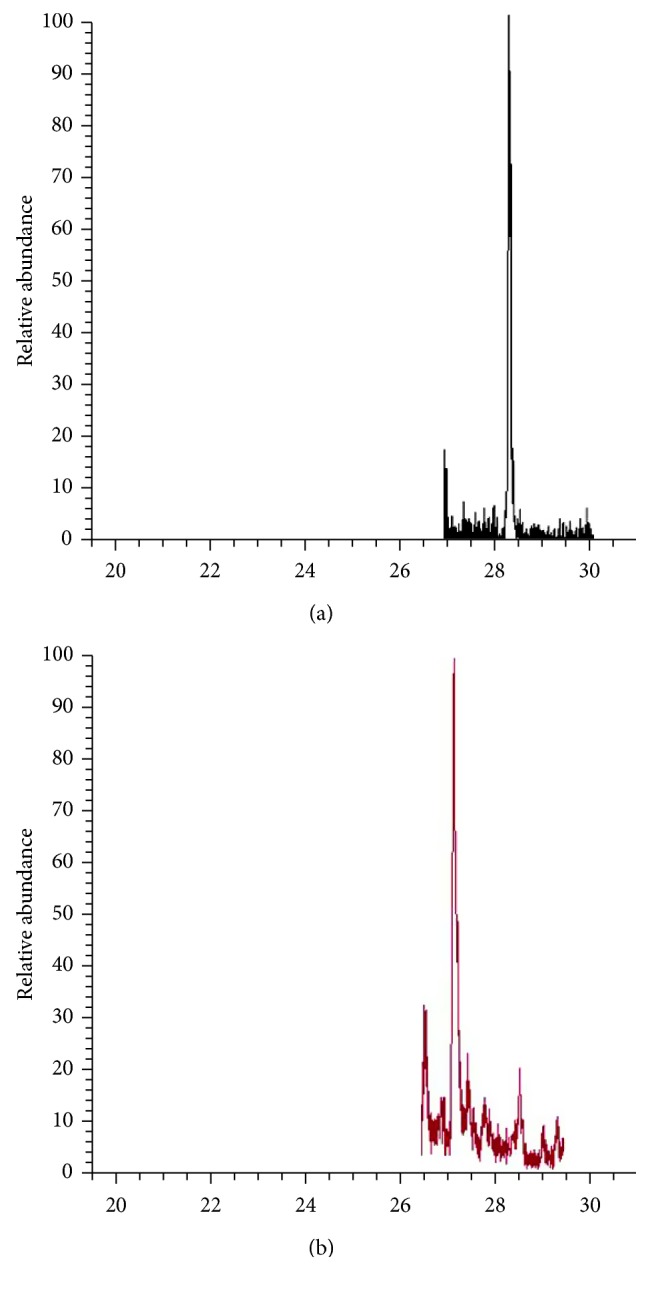
False-positive detection of diclofenac in wastewater. The calculated ion ratio, 0.15, falls outside the ion ratio tolerance range of 1.57 to 3.67. The chromatograms show (a) the peak for the quantitative ion with a transition of 296 → 214 and (b) the peak for the qualitative ion with a transition of 296 → 250.

An example of a false-positive detection that was found through the use of the ion ratio is diclofenac. The quantitative and qualitative ion transitions of diclofenac are 296 → 214 and 296 → 250 and its retention time is at 27.5 min.  Figure 5 shows a comparison of the two chromatograms, both of which have peaks at 27 min for both SRM transitions.

When the ion ratios were calculated, an influent sample (Figure 5) gave an ion ratio of 0.15, which falls outside the range for diclofenac in the influent which is from 1.57 to 3.67. Furthermore, it can be observed that, for WWTP A, the retention time of the qualitative ion, which is at 27.16 min, is slightly different from that of the quantitative one at 27.78 min, further proving that this is a false-positive detection since the retention times of both ions should be the same. Upon removal of 7 false-positive diclofenac peaks that did not match the ion ratio criterion and retention times for both the quantitative and qualitative ions, the total number of detections was reduced from 312 to 305.

## 4. Conclusions

An ion ratio criterion has been optimized for six classes of pharmaceuticals in wastewater and surface water using LC-MS/MS. For 23 PPCPs, values for mean ± tolerance for the ion ratios in the different types of environmental matrices were established based on the variabilities of the ion ratios in spiked samples. The variabilities of the ion ratios of the compounds were found to increase at lower concentrations from 4% at 100 ppb to 18% at 1.56 ppb. Therefore, the mean ion ratio that was used in the formula is the average of the ion ratios from 1.56, 3.13, 6.25, 12.5, 25, 50, and 100 ppb so that it can capture the variations at different concentrations. The ion ratios for tetracyclines and quinolones were found to have higher variations, which are twice that of the other PPCPs; therefore, these two classes of compounds were analyzed separately so as not to increase the possibility of false positives for the other compound classes. For tetracyclines and quinolones, the tolerance range was set to 80%, but it is recommended that other criteria such as retention time, peak areas, and the number of points per peak be investigated carefully before reporting their detections.

For the sulfonamides, macrolides, SSRIs, and other PPCPs, the ion ratios were studied in the different environmental matrices. It was found that the variations also increase with the complexity of the matrix. The optimized tolerance range that would give <5% false negatives was 40% for the influent and 30% for the effluent, upstream, and downstream. This optimized ion ratio criterion was then applied to real wastewater and surface water samples that were not spiked with standards and resulted in the reduction of the total number of detections from 312 to 305, after false positives were eliminated.

## Figures and Tables

**Table 1:** Target pharmaceuticals used for establishing the ion ratio criterion.

Class	Compound
*Antibiotics*	
Macrolides	Anhydroerythromycin
	Azithromycin
	Clarithromycin

Quinolones	Ciprofloxacin
	Enrofloxacin
	Norfloxacin
	Sarafloxacin

Sulfonamides	Acetylsulfamethoxazole
	Sulfachloropyridazine
	Sulfadiazine
	Sulfadimethoxine
	Sulfamerazine
	Sulfamethazine
	Sulfamethizole
	Sulfamethoxazole
	Sulfamethoxydiazine
	Sulfathiazole

Tetracyclines	Chlortetracycline
	Oxytetracycline
	Tetracycline

*Other PPCPs*	
	Acetaminophen
	Caffeine
	Carbamazepine
	Diclofenac
	Iopamidol
	Trimethoprim
	Bupropion
	Paroxetine
	Sertraline
	Venlafaxine

**Table 2:** Percent false negatives in spiked environmental matrices at different tolerance ranges for all 30 PPCPs.

Tolerance range	Matrix				Overall false negatives
	Influent	Effluent	Downstream	Upstream	
±10%	54%	49%	47%	42%	38%
±20%	26%	20%	16%	14%	16%
±30%	13%	9%	7%	6%	8%
±40%	8%	6%	4%	3%	6%
±50%	5%	4%	2%	2%	4%

**Table 3:** Percent false negatives in spiked environmental matrices at different tolerance ranges for 23 compounds without tetracyclines and quinolones.

Tolerance range	Matrix				Overall false negatives
	Influent	Effluent	Downstream	Upstream	
±10%	48%	42%	41%	35%	31%
±20%	20%	13%	10%	8%	10%
±30%	8%	4%	2%	2%	4%
±40%	4%	2%	0%	0%	2%
±50%	2%	1%	0%	0%	1%

**Table 4:** Percent false negatives for tetracyclines and quinolones at the 50% tolerance range in spiked environmental matrices.

Compounds	Matrix				Overall false negatives
	Influent	Effluent	Downstream	Upstream	
Chlortetracycline	10%	0%	0%	0%	2%
Ciprofloxacin	96%	71%	33%	13%	61%
Enrofloxacin	5%	0%	0%	0%	1%
Norfloxacin	0%	4%	0%	0%	1%
Oxytetracycline	0%	30%	29%	22%	21%
Sarafloxacin	12%	16%	0%	20%	13%
Tetracycline	11%	0%	0%	0%	3%

**Table 5:** Percent false negatives for tetracyclines and quinolones in spiked environmental matrices at the 80% tolerance range.

Compounds	Matrix				Overall false negatives
	Influent	Effluent	Downstream	Upstream	
Chlortetracycline	10%	0%	0%	0%	2%
Ciprofloxacin	0%	0%	0%	0%	0%
Enrofloxacin	0%	0%	0%	0%	0%
Norfloxacin	0%	4%	0%	0%	1%
Oxytetracycline	0%	0%	29%	0%	6%
Sarafloxacin	6%	11%	0%	20%	10%
Tetracycline	0%	0%	0%	0%	0%

**Table 6:** Results of the application of the ion ratio criterion in real wastewater and surface water samples.

Matrix	Tolerance range	Total no. of detections	No. of detections outside the range
Influent	±40%	102	0
Effluent	±30%	90	2
Downstream	±30%	37	2
Upstream	±30%	39	1

A number of detections outside the range are data points that were considered positive detections but had ion ratios outside the set tolerance range.

**Table 7:** Compounds with ion ratios detected outside the set tolerance range of ±40% for WWTP influent samples and 30% for WWTP effluent, upstream, and downstream samples.

Matrix	Compound	Tolerance range	Calculated ion ratio
Effluent	Acetylsulfamethoxazole	0.88–1.64	1.74
	Azithromycin	1.89–3-5	1.86

Downstream	Clarithromycin	1.28–2.38	2.41
	Clarithromycin	1.28–2.38	2.42

Upstream	Azithromycin	1.89–3-5	1.74

## Data Availability

All data underlying the findings of this study can be accessed in the supplementary information provided.

## Supplementary Materials

Supplementary Materials Table S1: retention time and transitions of pharmaceuticals in the study. Table S2: average ion ratios of pharmaceuticals at different concentrations.

## References

[1] Arnnok P., Singh R. R., Burakham R., Pérez-Fuentetaja A., Aga D. S. (2017). Selective uptsake and bioaccumulation of antidepressants in fish from effluent-impacted Niagara River. *Environmental Science and Technology*.

[2] Kummerer K. (2009). Antibiotics in the aquatic environment–a review–part I. *Chemosphere*.

[3] Senta I., Krizman-Matasic I., Terzic S., Ahel M. (2017). Comprehensive determination of macrolide antibiotics, their synthesis intermediates and transformation products in wastewater effluents and ambient waters by liquid chromatography-tandem mass spectrometry. *Journal of Chromatography A*.

[4] Senta I., Terzić S., Ahel M. (2008). Simultaneous determination of sulfonamides, fluoroquinolones, macrolides and trimethoprim in wastewater and river water by LC-tandem-MS. *Chromatographia*.

[5] Zhou L. J., Ying G. G., Liu S. (2013). Occurrence and fate of eleven classes of antibiotics in two typical wastewater treatment plants in South China. *Science of the Total Environment*.

[6] Pedrouzo M., Borrull F., Marce R. M., Pocurull E. (2009). Ultra-high-performance liquid chromatography-tandem mass spectrometry for determining the presence of eleven personal care products in surface and wastewaters. *Journal of Chromatography A*.

[7] Kristiansson E., Fick J., Janzon A. (2011). Pyrosequencing of antibiotic-contaminated river sediments reveals high levels of resistance and gene transfer elements. *PLoS one*.

[8] Rizzo L., Manaia C., Merlin C. (2013). Urban wastewater treatment plants as hotspots for antibiotic resistant bacteria and genes spread into the environment: a review. *Science of the Total Environment*.

[9] Kümmerer K., Henninger A. (2003). Promoting resistance by the emission of antibiotics from hospitals and households into effluent. *Clinical Microbiology and Infection*.

[10] Tran N. H., Reinhard M., Gin K. Y. H. (2018). Occurrence and fate of emerging contaminants in municipal wastewater treatment plants from different geographical regions-a review. *Water Research*.

[11] WHO (2017). *Antimicrobial Resistance*.

[12] Schoenfuss H. L., Furlong E. T., Phillips P. J. (2016). Complex mixtures, complex responses: assessing pharmaceutical mixtures using field and laboratory approaches. *Environmental Toxicology and Chemistry*.

[13] Tran N. H., Chen H., Reinhard M., Mao F., Gin K. Y. (2016). Occurrence and removal of multiple classes of antibiotics and antimicrobial agents in biological wastewater treatment processes. *Water Research*.

[14] Berendsen B. J., Meijer T., Mol H. G., van Ginkel L., Nielen M. W. (2017). A global inter-laboratory study to assess acquisition modes for multi-compound confirmatory analysis of veterinary drugs using liquid chromatography coupled to triple quadrupole, time of flight and orbitrap mass spectrometry. *Analytica Chimica Acta*.

[15] FDA (2003). *Guidance for Industry 118 Confirmation of Identity of Animal Drug Residues*.

[16] UNODC (2009). *Guidance for the Validation of Analytical Methodology and Calibration of Equipment Used for Testing of Illicit Drugs in Seized Materials and Biological Specimens*.

[17] The European Communities (2002). *Commission Decision 2002/657/EC Implementing Council Directive 96/23/EC Concerning the Performance of Analytical Methods and the Interpretation of Results*.

[18] USDA (2017). *Data and Instrumentation Revision 5. Agricultural Marketing Service*.

[19] EWDTS (2002). *European Laboratory Guidelines for Legally Defensible Workplace Drug Testing (EWDTS)*.

[20] Mol H. G., Zomer P., Garcia Lopez M. (2015). Identification in residue analysis based on liquid chromatography with tandem mass spectrometry: experimental evidence to update performance criteria. *Analytica Chimica Acta*.

[21] Berendsen B. J., Meijer T., Wegh R. (2016). A critical assessment of the performance criteria in confirmatory analysis for veterinary drug residue analysis using mass spectrometric detection in selected reaction monitoring mode. *Drug Testing and Analysis*.

[22] USEPA (2007). *Method 1694: Pharmaceuticals and Personal Care Products in Water, Soil, Sediment, and Biosolids by HPLC/MS/MS*.

[23] USEPA (2016). *Method 542: Determination of Pharmaceuticals and Personal Care Products in Drinking Water by Solid Phase Extraction and Liquid Chromatography Electrospray Ionization–Tandem Mass Spectrometry (LC/ESI-MS/MS)*.

