# Supplementary material for: Establishing Analytical Performance Criteria for the Global Reconnaissance of Antibiotics and Other Pharmaceutical Residues in the Aquatic Environment Using Liquid Chromatography-Tandem Mass Spectrometry
Source: J Anal Methods Chem. 2018 Jun 4;2018:7019204. doi: 10.1155/2018/7019204 (PMC6008649; doi:10.1155/2018/7019204)
Supplement: Supplementary Materials — Table S1: retention time and transitions of pharmaceuticals in the study. Table S2: average ion ratios of pharmaceuticals at different concentrations. [file 7019204.f1.docx]

**Establishing Analytical Performance Criteria for the Global Reconnaissance of Pharmaceutical Residues in the Aquatic Environment using Liquid chromatography/mass spectrometry**

Luisa F. Angeles^1^ and Diana S. Aga^1^

^1^Chemistry Department, University at Buffalo, The State University of New York, Buffalo, NY USA 14226

Correspondence: Diana S. Aga; dianaaga@buffalo.edu

Table S1. Retention time and transitions of pharmaceuticals in the study

| **Compounds** | **Retention Time** | **Precursor Ion** | **Tube Lens** | **Fragment 1 (CE)** | **Fragment 2 (CE)** |
| --- | --- | --- | --- | --- | --- |
| MACROLIDES |  |  |  |  |  |
| anhydro erythromycin | 20.67 | 716.5 | 89 | 158 (28) | 558.4 (16) |
| azithromycin | 15.67 | 749.5 | 116 | 157.9 (36) | 591.4 (25) |
| clarithromycin | 21.68 | 748.5 | 105 | 157.9 (26) | 590.5 (16) |
| PPCPs |  |  |  |  |  |
| acetaminophen | 5.73 | 152.1 | 59 | 65.1 (30) | 110.1 (15) |
| caffeine | 13.76 | 195.1 | 71 | 110 (25) | 138.1 (19) |
| carbamazepine | 21.86 | 237.1 | 97 | 193.1 (33) | 194.1 (19) |
| diclofenac | 27.5 | 296 | 53 | 214 (33) | 250 (13) |
| iopamidol | 2.55 | 777.9 | 142 | 386.9 (40) | 558.9 (22) |
| trimethoprim | 9.08 | 291.1 | 94 | 230.1 (23) | 261.1 (24) |
| QUINOLONES |  |  |  |  |  |
| ciprofloxacin | 13.58 | 332.1 | 78 | 231 (35) | 288.1 (16) |
| enrofloxacin | 14.13 | 360.2 | 81 | 245.1 (25) | 316.2 (18) |
| norfloxacin | 13.18 | 320.1 | 70 | 233.1 (23) | 276.1 (16) |
| sarafloxacin | 14.95 | 386.1 | 85 | 299.1 (26) | 342.1 (17) |
| SSRIs |  |  |  |  |  |
| bupropion | 15.3 | 240.1 | 58 | 131.1 (27) | 184 (12) |
| paroxetine | 19.42 | 330.2 | 100 | 150.9 (23) | 192.1 (20) |
| sertraline | 21.05 | 306.1 | 46 | 158.9 (28) | 275 (11) |
| venlafaxine | 16.19 | 278.2 | 67 | 58.2 (17) | 121.1 (29) |
| SULFONAMIDES |  |  |  |  |  |
| acetylsulfamethoxazole | 18.01 | 296.1 | 73 | 134 (24) | 198 (17) |
| sulfachloropyrazidine | 15.54 | 285 | 61 | 108 (25) | 156 (15) |
| sulfadiazine | 6.82 | 251.1 | 60 | 92.1 (28) | 156 (15) |
| sulfadimethoxine | 18.74 | 311.1 | 73 | 108 (30) | 156 (21) |
| sulfamerazine | 10.78 | 265.1 | 65 | 156 (16) | 172 (17) |
| sulfamethazine | 13.33 | 279.1 | 70 | 124.1 (26) | 186 (17) |
| sulfamethizole | 14.07 | 271 | 60 | 92.1 (27) | 156 (14) |
| sulfamethoxazole | 16.03 | 254 | 54 | 92.1 (26) | 156 (16) |
| sulfamethoxydiazine | 13.98 | 281.1 | 65 | 92 (29) | 108 (26) |
| sulfathiazole | 8.96 | 256 | 59 | 92 (27) | 156 (15) |
| TETRACYCLINES |  |  |  |  |  |
| chlorotetracycline | 15.6 | 479.1 | 88 | 444.1 (20) | 462.2 (16) |
| oxytetracycline | 12.35 | 461.1 | 71 | 337.1 (28) | 426.1 (71) |
| tetracycline | 7.82 | 445.2 | 105 | 154 (25) | 410.2 (17) |

Table S2. Average ion ratios of pharmaceuticals at different concentrations

| **Compounds** | **Average Ion Ratio** | | | | | | | |
| --- | --- | --- | --- | --- | --- | --- | --- | --- |
|  | **Overall** | **1.56** | **3.13** | **6.25** | **12.5** | **25** | **50** | **100** |
| MACROLIDES |  |  |  |  |  |  |  |  |
| anhydro erythromycin | 1.15 | 1.24 | 1.37 | 1.12 | 1.12 | 1.17 | 1.00 | 1.00 |
| azithromycin | 2.69 | 3.16 | 2.68 | 2.62 | 2.47 | 2.55 | 2.72 | 2.66 |
| clarithromycin | 1.83 | 2.22 | 1.77 | 1.87 | 1.74 | 1.78 | 1.72 | 1.70 |
| PPCPs |  |  |  |  |  |  |  |  |
| acetaminophen | 4.74 | 5.16 | 4.86 | 4.78 | 4.62 | 4.62 | 4.54 | 4.61 |
| caffeine | 5.29 | 5.02 | 5.59 | 5.08 | 5.26 | 5.47 | 5.36 | 5.24 |
| carbamazepine | 3.24 | 3.25 | 3.25 | 3.25 | 3.25 | 3.24 | 3.25 | 3.24 |
| diclofenac | 2.62 | 2.61 | 2.69 | 2.64 | 2.65 | 2.62 | 2.58 | 2.58 |
| iopamidol | 2.90 | 3.52 | 3.25 | 3.01 | 2.85 | 2.66 | 2.52 | 2.52 |
| trimethoprim | 0.77 | 0.77 | 0.78 | 0.74 | 0.76 | 0.77 | 0.76 | 0.78 |
| QUINOLONES |  |  |  |  |  |  |  |  |
| ciprofloxacin | 4.61 | 3.75 | 4.40 | 5.11 | 4.63 | 5.20 | 4.60 | 4.54 |
| enrofloxacin | 2.41 |  | 2.19 | 2.91 | 2.84 | 2.25 | 2.15 | 2.12 |
| norfloxacin | 1.31 |  |  | 1.17 | 1.20 | 1.35 | 1.37 | 1.46 |
| sarafloxacin | 1.10 |  |  | 1.05 | 1.14 | 1.15 | 1.06 | 1.07 |
| SSRIs |  |  |  |  |  |  |  |  |
| bupropion | 1.73 | 1.74 | 1.77 | 1.72 | 1.71 | 1.74 | 1.74 | 1.73 |
| paroxetine | 5.06 | 5.48 | 5.10 | 4.96 | 4.99 | 5.03 | 4.93 | 4.95 |
| sertraline | 1.17 | 1.22 | 1.14 | 1.19 | 1.17 | 1.17 | 1.16 | 1.14 |
| venlafaxine | 1.61 | 1.62 | 1.65 | 1.63 | 1.62 | 1.60 | 1.62 | 1.58 |
| SULFONAMIDES |  |  |  |  |  |  |  |  |
| acetylsulfamethoxazole | 1.26 | 1.37 | 1.25 | 1.26 | 1.25 | 1.26 | 1.22 | 1.23 |
| sulfachloropyrazidine | 2.52 | 2.75 | 2.65 | 2.53 | 2.36 | 2.42 | 2.45 | 2.44 |
| sulfadiazine | 2.16 | 2.44 | 2.21 | 2.16 | 2.10 | 2.04 | 2.05 | 2.08 |
| sulfadimethoxine | 3.52 | 3.60 | 3.57 | 3.46 | 3.57 | 3.53 | 3.46 | 3.43 |
| sulfamerazine | 1.06 | 1.09 | 1.06 | 1.04 | 1.07 | 1.07 | 1.06 | 1.04 |
| sulfamethazine | 3.01 | 3.12 | 3.08 | 3.04 | 3.04 | 2.95 | 2.91 | 2.89 |
| sulfamethizole | 0.44 | 0.48 | 0.40 | 0.45 | 0.43 | 0.44 | 0.45 | 0.44 |
| sulfamethoxazole | 1.47 | 1.64 | 1.45 | 1.47 | 1.46 | 1.42 | 1.44 | 1.43 |
| sulfamethoxydiazine | 0.87 | 0.87 | 0.85 | 0.86 | 0.88 | 0.86 | 0.89 | 0.88 |
| sulfathiazole | 2.61 | 2.89 | 2.78 | 2.50 | 2.48 | 2.56 | 2.52 | 2.57 |
| TETRACYCLINES |  |  |  |  |  |  |  |  |
| chlorotetracycline | 0.69 |  |  |  | 0.59 | 0.66 | 0.74 | 0.77 |
| oxytetracycline | 5.86 |  |  |  |  | 5.14 | 6.50 | 5.86 |
| tetracycline | 28.66 |  |  |  | 38.80 | 30.19 | 26.53 | 20.58 |
